# Supplementary material for: Modeling glioblastoma heterogeneity as a dynamic network of cell states
Source: Mol Syst Biol. 2021 Sep 16;17(9):e10105. doi: 10.15252/msb.202010105 (PMC8444284; doi:10.15252/msb.202010105)
Supplement: Supplementary file 6 — Source Data for Figure 5 [file MSB-17-e10105-s004.zip › Figure5A_sourcedata/GSEA_3017/hallmarks_stateD.GseaPreranked.1621934542601/HALLMARK_APOPTOSIS.html]

Details for gene set HALLMARK\_APOPTOSIS[GSEA]

|  || Dataset | state23017 |
| Phenotype | NoPhenotypeAvailable |
| Upregulated in class | na\_neg |
| GeneSet | HALLMARK\_APOPTOSIS |
| Enrichment Score (ES) | -0.45741192 |
| Normalized Enrichment Score (NES) | -1.932846 |
| Nominal p-value | 0.005611672 |
| FDR q-value | 0.03799054 |
| FWER p-Value | 0.1 |
Table: GSEA Results Summary

  

Fig 1: Enrichment plot: HALLMARK\_APOPTOSIS      
 Profile of the Running ES Score & Positions of GeneSet Members on the Rank Ordered List

  

| PROBE | GENE SYMBOL | GENE\_TITLE | RANK IN GENE LIST | RANK METRIC SCORE | RUNNING ES | CORE ENRICHMENT || 1 | HSPB1 |  |  | 52 | 0.347 | -0.0165 | No |
| 2 | VDAC2 |  |  | 114 | 0.279 | -0.0445 | No |
| 3 | MCL1 |  |  | 115 | 0.279 | -0.0254 | No |
| 4 | IFNGR1 |  |  | 288 | -0.263 | -0.1405 | No |
| 5 | DNAJC3 |  |  | 366 | -0.269 | -0.1816 | No |
| 6 | BNIP3L |  |  | 431 | -0.277 | -0.2122 | No |
| 7 | CASP3 |  |  | 459 | -0.280 | -0.2139 | No |
| 8 | CDC25B |  |  | 472 | -0.282 | -0.2038 | No |
| 9 | BTG3 |  |  | 581 | -0.296 | -0.2671 | No |
| 10 | APP |  |  | 626 | -0.306 | -0.2802 | No |
| 11 | FDXR |  |  | 703 | -0.320 | -0.3171 | No |
| 12 | TIMP3 |  |  | 784 | -0.339 | -0.3557 | No |
| 13 | CDKN1B |  |  | 862 | -0.359 | -0.3907 | No |
| 14 | CFLAR |  |  | 873 | -0.364 | -0.3735 | No |
| 15 | TGFB2 |  |  | 903 | -0.376 | -0.3702 | No |
| 16 | DPYD |  |  | 909 | -0.377 | -0.3482 | No |
| 17 | NEDD9 |  |  | 993 | -0.412 | -0.3842 | No |
| 18 | BAX |  |  | 1032 | -0.434 | -0.3838 | No |
| 19 | SAT1 |  |  | 1109 | -0.473 | -0.4102 | No |
| 20 | BRCA1 |  |  | 1171 | -0.541 | -0.4203 | Yes |
| 21 | CLU |  |  | 1207 | -0.592 | -0.4068 | Yes |
| 22 | ANXA1 |  |  | 1221 | -0.623 | -0.3742 | Yes |
| 23 | MMP2 |  |  | 1260 | -0.733 | -0.3534 | Yes |
| 24 | HMGB2 |  |  | 1263 | -0.736 | -0.3044 | Yes |
| 25 | CD44 |  |  | 1270 | -0.762 | -0.2568 | Yes |
| 26 | IFITM3 |  |  | 1279 | -0.812 | -0.2073 | Yes |
| 27 | LGALS3 |  |  | 1287 | -0.878 | -0.1525 | Yes |
| 28 | CDKN1A |  |  | 1306 | -1.046 | -0.0947 | Yes |
| 29 | TOP2A |  |  | 1313 | -1.528 | 0.0054 | Yes |
Table: GSEA details [plain text format]

  

Fig 2: HALLMARK\_APOPTOSIS: Random ES distribution      
 Gene set null distribution of ES for **HALLMARK\_APOPTOSIS**
